# Supplementary material for: CRISPR-Cas9-based repeat depletion for high-throughput genotyping of complex plant genomes
Source: Genome Res. 2023 May;33(5):787–97. doi: 10.1101/gr.277628.122 (PMC10317117; doi:10.1101/gr.277628.122)
Supplement: Supplemental Material [file supp_33_5_787__DC1.html]

CRISPR-Cas9-based repeat depletion for high-throughput genotyping of complex plant genomes — Supplemental Material 

# CRISPR-Cas9-based repeat depletion for high-throughput genotyping of complex plant genomes

## Supplemental Material

- Supplemental\_File\_S1.xlsx
- Supplemental\_Tables\_Revised.xlsx
- Supplemental\_Figures\_Revised.docx
